# Do people from the Jewish community prefer ancestry-based or pan-ethnic expanded carrier screening?

**DOI:** 10.1038/ejhg.2015.97

**Published:** 2015-05-13

**Authors:** Kim C A Holtkamp, Merel C van Maarle, Maria J E Schouten, Wybo J Dondorp, Phillis Lakeman, Lidewij Henneman

**Affiliations:** 1 Department of Clinical Genetics, Section of Community Genetics, EMGO Institute for Health and Care Research, VU University Medical Center, Amsterdam, The Netherlands; 2 Department of Clinical Genetics, Academic Medical Center, Amsterdam, The Netherlands; 3 Department of Health, Ethics and Society, Research Institutes CAPHRI and GROW, Maastricht University, Maastricht, The Netherlands

## Abstract

Ancestry-based carrier screening in the Ashkenazi Jewish population entails screening for specific autosomal recessive founder mutations, which are rarer among the general population. As it is now technically feasible to screen for many more diseases, the question arises whether this population prefers a limited ancestry-based offer or a pan-ethnic expanded carrier screening panel that goes beyond the diseases that are frequent in their own population, and is offered regardless of ancestry. An online questionnaire was completed by 145 individuals from the Dutch Jewish community (≥18 years) between April and July 2014. In total, 64.8% were aware of the existence of ancestry-based carrier screening, and respondents were generally positive about screening. About half (53.8%) preferred pan-ethnic expanded carrier screening, whereas 42.8% preferred ancestry-based screening. Reasons for preferring pan-ethnic screening included ‘everyone has a right to be tested', ‘fear of stigmatization when offering ancestry-based panels', and ‘difficulties with identifying risk owing to mixed backgrounds'. ‘Preventing high healthcare costs' was the most important reason against pan-ethnic carrier screening among those in favor of an ancestry-based panel. In conclusion, these findings show that people from the Dutch Jewish community have a positive attitude regarding carrier screening in their community for a wide range of diseases. As costs of expanded carrier screening panels are most likely to drop in the near future, it is expected that these panels will receive more support in the future.

## Introduction

The Ashkenazi Jewish (AJ) population is at increased risk for several autosomal recessive diseases, such as Tay–Sachs disease (TSD) and Canavan disease, owing to genetic drift and founder effect.^1^ Worldwide, sections of the AJ population have been well familiar with ancestry-based carrier screening since the 1970s. This form of carrier screening entails screening for specific founder mutations corresponding with several severe autosomal recessive diseases that are relatively common in the AJ population but that are rarer among the general population. Positive attitudes in the AJ population have been reported since the time screening became available.^2, 3, 4, 5^ In the ultra-orthodox AJ community, there is in particular experience with a premarital confidential carrier matching program (Dor Yeshorim),^2^ where screening is performed without disclosure of individual test results and couples are only told whether they are ‘compatible' or not. In the more liberal and less orthodox communities, screening is offered with the aim of enhancing reproductive decision making among identified carrier couples, who face a risk of 1 in 4 of having an affected child at every conception. Other programs include screening adolescents in high schools.^5^

While in the 1970s carrier screening was offered for TSD only, resulting in a 90% decrease in the number of cases,^6, 7^ ancestry-based carrier screening panels, available both commercially and via public health systems, have now expanded to include a wider range of diseases.^8, 9^ Despite recommendations by the American College of Obstetricians and Gynaecologists, and the American College of Medical Genetics,^10, 11^ these panels increasingly include less common and less severe diseases as well, but are nevertheless well accepted by the community.^12, 13^ It has been remarked that the expansion of these ancestry-based panels is partly driven by the community itself, especially by those who have personal experience with less common diseases that were not included in the earlier panels.^12^ Despite this latter aspect, the fact that these ancestry-based screening panels target a specific group is also associated with concerns about a higher perceived risk of stigmatization and perceived vulnerability.^13, 14^

Because of the technological advances, it is now possible to simultaneously screen for carrier status for many more autosomal recessive diseases.^15^ The question arises whether the AJ population prefers an explicit and limited ancestry-based offer or an expanded carrier screening panel, offered to the entire population. This expanded panel will also include other recessive diseases besides those that are more frequent among Ashkenazi Jews, which can thereby be offered ‘pan-ethnically' (universally).

Approximately 37 000–53 000 Jews live in the Netherlands, 90–95% of whom are of AJ descent.^16^ The Netherlands is a country with relatively little experience of carrier screening for AJ founder mutations, and little is known about experiences with carrier screening among individuals from the Dutch AJ community. Although preconception carrier screening for AJ couples with no family history of disease is available in at least two Dutch University hospitals and reimbursed by most healthcare insurance companies, very few couples actually request testing. Results from a pilot interview study with nine community members suggest that people often go abroad to be tested (eg, United States, United Kingdom, and Israel) owing to unfamiliarity with the availability of testing in the Netherlands (unpublished results). This might indicate a need for awareness and a more active offer of carrier screening in the Netherlands. The question of how this should be offered, taking into account the preferences of the target population, is the subject of this study.

The following research questions were addressed: (1) What are the attitudes and intentions of the Dutch AJ community towards carrier screening aimed at severe genetic diseases that are more common in the Jewish community?; (2) Do they prefer an ancestry-based or pan-ethnic expanded carrier screening panel, and why?; (3) Which categories of diseases should be included in a carrier screening panel?; and (4) How should carrier screening be offered (ie, timing, setting, financing, and test results disclosure)?

## Materials and Methods

### Respondents and procedures

From April to July 2014, individuals of Jewish ancestry (≥18 years) living in the Netherlands were invited to complete an online questionnaire, available in English and Dutch. Respondents were recruited in four different ways. First, after consultation and with consent of two rabbis, the questionnaire was placed on the websites of the Liberal Jewish Community in Amsterdam (~1800 members), and of the Dutch Israelite religious community (NIK; ~4000 members nationwide). Second, the link to the questionnaire was placed in the (online) newsletter of the NIK as well as in the national New Israelite Weekly Journal (print run of 5500). Third, a midwifery practice in a city with a relatively large Jewish community posted the link on their website. Finally, respondents were recruited via snowball sampling:^17^ a mailing in English and Dutch with the link to the questionnaire was sent to five key contacts within the Jewish community, which were established through contact with two rabbis, and the researchers' network. The Medical Ethical Committee of VU University Medical Center Amsterdam approved the study protocol.

### Survey instrument

The questionnaire was developed specifically for this study by the members of the research team (two clinical geneticists, two health scientists, and an ethicist), and based on nine exploratory interviews, and the literature. People who participated had a chance of winning a €25 gift voucher.

The questionnaire (Supplementary Materials and Methods) first explained the concept of carrier screening. Respondents were asked if, before receiving the questionnaire, they had heard about the existence of carrier screening for diseases relatively common in the Jewish community, if they had been tested before, and if so where. In addition, respondents were asked whether they knew someone with a severe genetic disease. The other topics investigated were: Attitude towards carrier screening in the Jewish community was measured using a semantic differential five-point scale with four bipolar adjective pairs: good–bad, alarming–not alarming, desirable–not desirable, and self-evident–not self-evident. The attitude statement was: ‘Offering carrier tests specifically aimed at severe genetic diseases that are more common in the Jewish community is…'. Intention was measured with a single item ‘Would you have a carrier test yourself?' (certainly (1) to certainly not (5)).

Specific questions addressed the following perceptions regarding carrier screening (Table 1): perceived benefits (3 statements); perceived social barriers (4 statements); worry (1 statement); and the directiveness of how carrier screening should be offered (3 statements). All items were answered on a five-point scale (completely disagree (1) to completely agree (5)).

One question assessed the preference of respondents regarding the offer of an ancestry-based versus a pan-ethnic expanded panel. It was first explained that although diseases such as TSD occur particularly in the AJ community, in general, all prospective parents, regardless of their ancestry, might give birth to a baby with a serious genetic disease. Respondents had to indicate their preference as following: (a) an ancestry-based panel: each subpopulation receives a different carrier test, which only tests for the common diseases in this group or (b) a pan-ethic expanded panel: regardless of origin, everyone in the Netherlands is offered the same carrier test, which tests for all possible genetic diseases. Then people could explain their answer in an open text box.

Categories of diseases to be included were evaluated by means of one question in which five categories of diseases were listed as following: (1) serious, life-threatening diseases for which no treatment is available; (2) diseases involving severe mental disability; (3) diseases involving a severe physical disability; (4) severe diseases that occur later in life; and (5) all diseases a couple wants to be tested for. Respondents were also asked whether or not couples should be given the free choice to decide for which of these categories of diseases they would like to be tested or whether the carrier screening panel should contain a closed list of diseases.

Furthermore, it was asked ‘how carrier screening should be offered'. Questions assessed the preferred timing of offering carrier screening (eg, via high schools, preconceptional, and prenatal), the preferred setting (eg, hospital, midwife, and internet), preferences regarding the financing of carrier screening (eg, reimbursement and how much respondents would be willing to pay), and preferences regarding disclosure of individual test results.

Finally, socio-demographic data including gender, age, level of education, (ancestral) origin, religiousness, relationship status, having children, planning to have children, and place of residence were collected.

### Data preparation and analysis

Descriptive analyses were used to describe respondents' characteristics. For the four bipolar adjective word pairs measuring attitude and the items measuring perceived benefits and perceived barriers, principle factor analysis with varimax rotation was used to assess possible subscales, followed by reliability analysis for internal consistency of the scales. This resulted in one attitude scale based on the mean ratings on the four word pairs (range 1–5; Cronbach's *α*=0.80), a perceived benefits scale (range 1–5; Cronbach's *α*=0.71), and a perceived social barriers scale (range 1–5; Cronbach's *α*=0.68). One item regarding partner choice did not fit any of the scales, and was analyzed separately. The two scales regarding perceived benefits and perceived social barriers as well as the single items listed were summarized to a three-point scale; (1) (completely) disagree (2) neither disagree nor agree, and (3) (completely) agree. A Mann–Whitney test was used to determine differences in attitude between liberal and orthodox Jews, and between people in the reproductive age group (18–45 years) and people older than 46 years (due to non-normality of these items). Differences in preferences for ancestry-based versus pan-ethnic carrier screening, and preferences regarding full disclosure of test results between liberal and orthodox Jews, and the two age groups were assessed by means of Pearson's *χ*^2^-test. Content analysis was used to analyze and categorize respondents' reasons for their preference regarding ancestry-based or pan-ethnic panels, given in the open text boxes. Statistical significance was set at *P<*0.05. All analyses were performed using IBM SPSS version 20 for Windows (IBM Corp, Armonk, NY, USA).

## Results

### Sample characteristics

**Figure 1:**
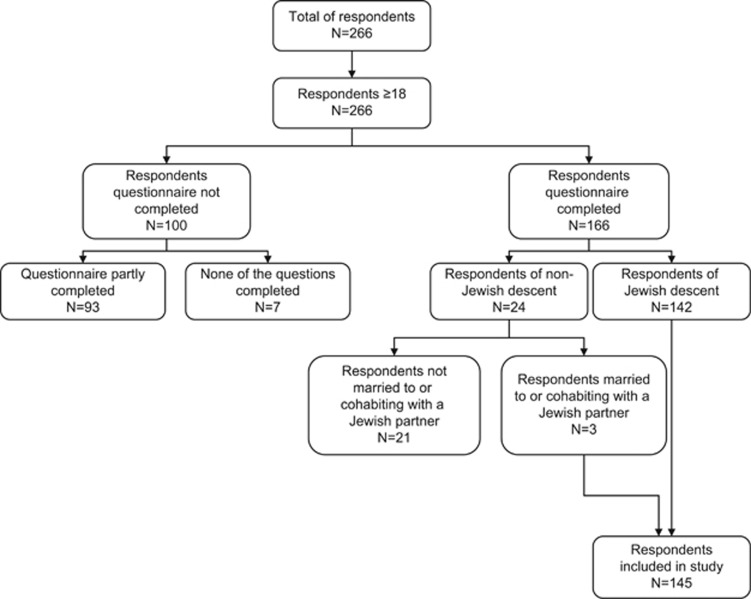
Flowchart inclusion of respondents.

In total, 266 respondents (all ≥18 years) responded, the majority of whom (*n*=166, 62.4%) completed all questions (Figure 1). It was not possible to trace via which of the four recruitment methods the respondents were included. Twenty-four people were initially excluded because of their non-Jewish ancestral background. Three of them, however, were in a relationship with a Jewish partner and were therefore included, resulting in a study sample of 145 respondents. People who fully completed the questionnaire had more often heard about carrier testing before they received the questionnaire than people who did not complete the questionnaire (‘non-completers') (85.3% versus 54.7%, respectively, *χ*^2^ (1)=5.52, *P*<0.05). As ‘non-completers' did not fill out the questions about socio-demographic variables, no comparison in characteristics between respondents and ‘non-completers' could be made.

Characteristics of respondents (*n*=145) are presented in Table 2. The majority were female (70.3%) with a mean age of 43 (range 18–87; SD=15.1). The mean age of the male respondents was 52 (range 20–76; SD=17.5). In total, 59.3% identified themselves as orthodox, either ultra or modern, and of all respondents, >85% were somewhat to very religiously active. Of all respondents with a partner (*n*=112), 47.4% (*n*=53) were considering a future pregnancy. Sixteen percent (*n*=23) had already been tested, of whom 11 were tested in Israel (10 by Dor Yeshorim), seven in the Netherlands, three in the US, one in the UK, and one in Greece. Finally, 64.8% had heard about carrier screening before receiving the questionnaire, and 41% knew someone with a severe genetic disease, not necessarily a genetic disease more common in the Jewish community.

### Attitudes and intention towards carrier screening

The majority had a positive attitude towards ancestry-based carrier screening in the Jewish community; 66.3% of the respondents scored a 4 or higher on the attitude scale (range 1–5). No differences in attitude were found between liberal (median (Mdn)=4.25) and orthodox Jews (Mdn=4.13), *U*=1420, *P*=0.112, *r*=0.14, nor between respondents from the reproductive age group (18–45 years; Mdn=4.25) or the older age group (Mdn=4.37), *U*=2261, *P*=0.165, *r*=−0.16. Twenty-nine of 51 respondents (56.9%) who were planning to have children and had not been tested before would certainly or probably want to have a carrier test, whereas 21.6% (*n*=11) doubted whether they would want to have a test, and 21.6% (*n*=11; probably) did not want to have a carrier test.

Respondents had high perceived benefits (Mdn=4.33; IQR=4.00–5.00) and low perceived social barriers (Mdn=2.25; IQR=1.50–2.75) regarding carrier screening in the Jewish community (Table 1). About one-third (29.7%) were worried about their own risk of being a carrier of a severe genetic disease. More than 90% agreed that Jews should be offered the option to have a carrier test. Although most respondents thought that Jewish couples were not obliged to get tested (56.6% agreed), 24.1% thought that healthcare professionals were allowed to insist on people getting tested.

### Preferences regarding an ancestry-based or pan-ethnic expanded panel

Overall, 53.8% of the respondents preferred pan-ethnic expanded carrier screening, whereas 42.8% preferred ancestry-based screening. Five people (3.4%) thought that carrier tests should not be offered at all (Table 3). The most frequently mentioned reason in favor of a pan-ethnic panel was ‘everyone has a right to be tested' (32.1%). Respondents indicated, for example, that everyone should have the right to have carrier testing for all disorders desired, and not only for the diseases with a higher ancestry-related risk. Furthermore, respondents thought that offering a pan-ethnic panel would be better because of ‘fear of stigmatization when offering ancestry-based panels' (18%), and ‘difficulties with identifying risks due to mixed backgrounds' (18%). ‘Preventing high healthcare costs' (33.9%) was the most important reason among those in favor of an ancestry-based panel, followed by ‘screening should better be based on high risk' (27.4%). No significant differences were found regarding the choice for an ancestry-based or pan-ethnic panel between liberal and orthodox Jews (*χ*^2^ (1)=0.30; *P*=0.58), and between the two age groups *χ*^2^ (1)=1.67; *P*=0.20.

### Categories of diseases to be included in a carrier screening panel

**Figure 2:**
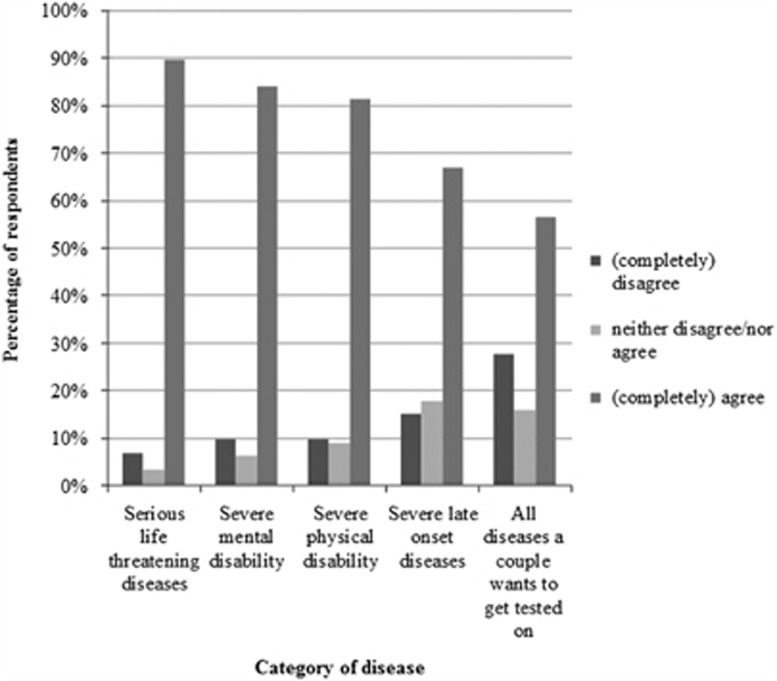
Preferences of respondents (*n*=145) regarding the categories of diseases to be included in a carrier screening panel.

Overall, the majority of the respondents thought all categories of diseases presented should be included in a panel (Figure 2). However, regarding less severe diseases, there was a slight variation in respondents' answers. Where 89.7% agreed that serious, life-threatening diseases should be included in a panel, 66.9% thought that severe late onset disorders should be included, and 56.6% agreed that carrier testing should include all diseases a couple wants to be tested for.

If a panel of carrier screening tests is offered to the AJ population, then 43.4% of the respondents thought that people should be able to decide themselves for what categories of disorders they wanted to be tested, and 36.6% preferred a carrier screening panel containing a closed list of diseases.

### How carrier screening should be offered

Most respondents thought that carrier screening should best be offered preconceptionally (29.9%), premaritally (24.1%), or to students (17.2%). Other options mentioned regarding timing were prenatal (14.9%), high school students (6.6%), new-born screening (3.4%), and other (3.7%). Hospitals (25.9%), GPs (24.6%), and midwifery practices (15.3%) were mentioned as the most appropriate settings for offering carrier screening, followed by Dor Yeshorim (14.3%), the rabbi (6.3%), Internet (5.0%), Jewish high schools (4.8%), or other (3.4%). The majority of the respondents (57.3%) were willing to pay 100–500 euros for screening, 25.5% were willing to pay <100 euros, whereas 17.1% were willing to pay >500 euros. Nevertheless, 57.9% thought that the costs should be reimbursed. Almost half of the respondents (46.2%) preferred only to be informed of their status as a couple, whereas 38.6% preferred full disclosure of their individual test results; 9.7% had no preference, and 3.5% thought it should be a couple's choice in deciding how to receive the test results. Orthodox Jews did not more often want to receive the test results as a couple compared with liberal Jews, *χ*^2^ (1)=1.20, *P*=0.27. In addition, no significant differences in preference were found between people from the reproductive age group (18–45 years) and people older than 46 years *χ*^2^ (1)=2.99, *P*=0.08.

## Discussion

This study suggests that people from the Dutch Jewish community are positive about carrier screening in their community, and that they generally perceive high benefits and low social barriers of carrier screening. Previous studies also show positive attitudes regarding carrier screening among people from AJ ancestry.^5, 18, 19^ Factors described to contribute to the high receptiveness of this community towards screening include the close involvement of the community, and consensus in favor of avoiding affected births.^20^ Furthermore, half of the respondents planning to have children in our study intended to have a carrier screening test. This finding is difficult to compare with other studies as most studies do not discuss intention but measure uptake in, for example, Dor Yeshorim or high school settings (eg, uptake rates over 94%).^5, 21, 22^ Though, as has been observed in different contexts, a gap exists between intention and actual behavior.^23^ It might therefore be expected that the actual uptake of carrier screening in the Netherlands will not be as high by far as the above-mentioned figures.

No convincing preference for ancestry-based or pan-ethnic carrier screening was shown. Slightly more than half of the respondents thought a pan-ethnic expanded panel was the best option. An important reason in favor of a pan-ethnic expanded panel was the fear of stigmatization when offering ancestry-based panels. Fear of stigmatization on both an individual level (eg, difficulties with finding a marriage partner within the community) and on a group level (the feeling that the entire community is at risk of being stigmatized as a result of ethnic categorization in screening) has been described before.^13, 14, 24^ Furthermore, about one-fifth of respondents who preferred a pan-ethnic expanded screening panel thought that because of mixed backgrounds it would be difficult to identify a person at risk in ancestry-based screening. This difficulty in determining risk groups was also mentioned by Jans *et al.*^25^ describing attitudes of GPs and midwives regarding ancestry-based haemoglobinopathy carrier screening. Moreover, it has been shown that many carriers are missed when conventional ancestry-based screening is used, because diseases also occur outside specific ethnic groups.^24^ ‘Increasing healthcare costs' was the most important reason among those in favor of an ancestry-based panel. Although it can be expected that the costs of pan-ethnic screening panels will decrease owing to advances in technology,^24^ some have argued that pan-ethnic expanded panels could conversely entail an increase in indirect costs (eg, costs of counseling),^26^ as more people will be identified as carriers. The question remains whether people's views on ancestry-based versus pan-ethnic screening would change if they knew that pan-ethnic carrier screening might become less expensive.

The majority of the respondents thought that all categories of diseases presented (from lethal in childhood to late onset diseases) should be included in a screening panel. Recent literature about the expansion of ancestry-based screening panels in the Jewish community observed that the expansion of these panels is largely driven by the community itself, and that there is support from the community to also include diseases that are less frequent, less detectable, and less severe.^12, 13^ As with all screening programs, expansion of screening panels should also be assessed in terms of accepted criteria for responsible screening.^27^ For instance, there is debate about whether expanded panels should also include lower-penetrance mutations, in which disease severity is difficult to predict and homozygotes may well remain asymptomatic. An example from current expanded ancestry-based panels in the Jewish community is type 1 Gaucher disease, which not only has a low-penetrance and variable expression but also has effective treatment available.^28^ It is important to at least be aware of the counseling challenges that may arise when offering screening panels containing different diseases with highly heterogeneous phenotypes, especially if the aim of the screening offer is to help people make well-informed reproductive decisions.

According to the Dutch Jewish community, carrier screening should preferentially be offered premaritally and preconceptionally by hospitals and GPs. Notably, a quarter of the respondents thought that health care professionals are allowed to insist on people getting tested. Respondents' preferences regarding the timing of offering carrier screening outside the pregnancy is in accordance with other literature, as this gives individuals more alternative reproductive choice and fewer time constraints.^2, 3, 22, 29^ Very few respondents felt that high schools should be the preferred setting for carrier screening, despite this setting being frequently discussed in the literature as an effective way to offer screening in the AJ community.^5, 21^ The low preference for this setting might be ascribed the fact that there are very few Dutch Jewish high schools and the lack of familiarity with this method of testing.

Almost half of the respondents preferred not to receive their individual test results and only wanted to be informed about whether they, as a couple, are at increased risk of having an affected child, whereas 38.6% wanted full disclosure of individual test results. This figure is much lower compared with carrier screening studies in other populations. Henneman *et al.*,^30^ for example, described that 94% of Dutch couples participating in preconceptional carrier screening for cystic fibrosis wanted full disclosure of test results, and only 4% wanted to receive results per couple. Different preferences regarding disclosure might be ascribed to cultural differences, and experiences with non-disclosure in the confidential carrier matching program Dor Yeshorim.

This is the first study describing the attitudes of people from the Dutch Jewish community towards carrier screening. Moreover, although studies have been conducted about the technical possibilities of expanding carrier screening panels in general, no previous research is known about preferences from target populations regarding their choice between ancestry-based and pan-ethnic expanded panels. It should be noted that this study has several limitations. For data collection, an online questionnaire was used, spread via snowball sampling and websites, which might have resulted in a selection bias. Little is known about people visiting these websites, and all respondent characteristics are self-reported. Furthermore, respondents with a higher education were over-represented, and this study has a large number of ‘non-completers'. Analysis showed that ‘non-completers' had heard less often about carrier screening previously than people who fully completed the questionnaire. This lack of familiarity with the topic as well as the relatively long questionnaire might have caused people to drop out, and caution is, therefore, required in generalizing the results. Finally, only few ultra-orthodox Jews, who may have different views, participated in this study.

In conclusion, our findings show that people from the Dutch Jewish community have a positive attitude regarding carrier screening in their community for a wide range of diseases. Although there was little consensus, there was a slight preference for pan-ethnic expanded carrier screening. The most important reason to prefer an ancestry-based panel was to prevent high costs. As costs of expanded carrier screening panels are most likely to drop in the near future, it is expected that these panels will receive more support in the future.

## Figures and Tables

**Table 1:** Distribution of agreement (*n* (%)) on statements regarding carrier screening in the Jewish community, *n*=145

		*(Completely) disagree*		*Neither disagree nor agree*		*(Completely) agree*	
*Scale/variables*	*Statements*	n	*%*	n	*%*	n	*%*
Perceived benefits	Offering a carrier test avoids much suffering	11	7.6	14	9.7	120	82.8
	A carrier test gives couples more certainty about their risk of having an affected child	9	6.2	8	5.5	128	88.3
	Carrier test results can help couples in making reproductive decisions about having children	11	7.6	19	13.1	115	79.3
Perceived social barriers	Offering a carrier test leads to anxiety in the Jewish community	82	56.6	31	21.4	32	22.1
	Offering a carrier test can cause people to feel forced to get tested	79	54.5	28	19.3	38	26.2
	Carrier testing will lead to carriers feeling left out of the Jewish community	102	70.3	24	16.6	19	13.1
	Offering a carrier test specifically aimed at the Jewish community leads to discrimination of Jews	109	75.2	15	10.3	21	14.5
Worry	I am worried about my own risk of being a carrier of a severe genetic disease	71	49.0	31	21.3	43	29.7
Partner choice	Carrier test results can help when choosing a partner	77	53.1	24	16.6	44	30.3
Directiveness of the offer	Every Jewish couple that wants to have children should have the option of having a carrier test	5	3.4	9	6.2	131	90.3
	Every Jewish couple that wants to have children is obliged to have a carrier test	82	56.6	29	20.0	34	23.4
	Healthcare professionals can force Jewish couples that want to have children to have a carrier test	88	60.7	22	15.2	35	24.1

**Table 2:** Characteristics of respondents, *n*=145

*Characteristics*	n	*%*
*Gender*		
Male	43	29.7
Female	102	70.3
		
*Age (years)*		
18–45	79	54.5
≥46	66	45.5
		
*Education*^a^ *(*n*=144)*		
Low/intermediate	27	18.8
High	117	81.2
		
*Ancestral origin*^b^ *(*n*=140)*		
Dutch	92	65.7
Western	30	21.4
Non-Western	18	12.9
		
*Origin*		
Ashkenazi Jewish descent	113	77.9
Sephardic Jewish descent	9	6.2
Mixed^c^	20	13.8
Non-Jewish descent^d^	3	2.1
		
*Religious affiliation*		
Ultra-orthodox Judaism	10	6.9
(Modern) orthodox Judaism	76	52.4
Liberal Judaism	40	27.6
None	15	10.3
Other	4	2.8
		
*Religious activity*		
Very active	52	35.9
Somewhat active	73	50.3
Not active/not applicable	20	13.8
		
*Relationship status*		
Married to/cohabiting with a Jewish partner	79	54.5
Married to/cohabiting with a non-Jewish partner	19	13.1
Single	41	28.3
Other^e^	6	4.1
		
*Having children*		
Yes	97	66.9
No	48	33.1
		
*Having a partner (*n*=112): planning to have (more) children*		
Yes/maybe	53	47.4
No/not applicable	59	52.6
		
*Place of residence (*n*=144)*		
Postal code region of Amsterdam	84	57.9
Other	60	41.4
		
*Have heard about carrier testing?*		
Yes	94	64.8
No	51	35.2
		
*Have you had a carrier test?*		
Yes	23	15.9
No	122	84.1

a Low: primary school, lower level of secondary school, lower vocational training. Intermediate: higher level of secondary school, intermediate vocational training. High: higher vocational training, university.

b Dutch, if both parents were born in the Netherlands; Western, if at least one of both parents was born in Europe (excluding Turkey), North-America, Oceania, Indonesia, or Japan; and Non-Western, if at least one of both parents was born in Africa, Latin-America, Asia (excluding Indonesia and Japan), and Turkey. If both parents were born abroad, then by country of mother.^31^

c Mixed includes: people from partly Ashkenazi/partly Sephardic descent, people from partly Ashkenazi/partly from non-Jewish descent, and people from partly Sephardic/partly non-Jewish descent.

d Married to/cohabiting with a Jewish partner.

e Other includes: engaged, non-cohabiting but with a partner.

**Table 3:** Preferences and reasons for preferring an expanded (*n*=78) or ancestry-based (*n*=62) carrier screening panel

*Preference*	*Reason*	*Example*	n	*%* a
Expanded panel (*n*=78; 53.8%)	Everyone has a right to be tested	‘Everyone should have the right to test whether he or she is a carrier, independent of background'	25	32.1
	Difficulties with identifying risk owing to mixed backgrounds	‘Subpopulations are not clearly divided any longer. There are too many mixed couples'	14	18.0
	Fear of stigmatization when offering ancestry-based panels	‘This (expanded panel) prevents stigmatization of communities'	14	18.0
	Avoiding inequity	‘Everyone would want the same certainty, Jewish or not Jewish'	10	12.8
	Targeted screening is not comprehensive enough	‘As the list of diseases is not exhaustive enough for each subpopulation, why not get tested on all diseases?'	8	10.3
	No specific reason		5	6.4
	Obtaining certainty	‘Everyone wants to know if he/she has a chance of having an affected child'	2	2.6
	Other		1	1.3
				
Ancestry-based panel (*n*=62; 42.8%)	Preventing high healthcare costs	‘I think that it is financially impossible to test everyone on all diseases'	21	33.9
	Screening should better be based on high risk	‘Why offer a test if the chance of having a disease is virtually nil?'	17	27.4
	No specific reason		10	16.1
	Most effective/most efficient	‘Testing everyone is unfeasible'	9	14.5
	All subgroups at risk should have a right to test	‘In my personal network, it is very common to test for these kinds of diseases. If there are other groups where there is also a predisposition for heritable diseases, they should have the right to test too'	5	8.1
	Other		3	4.8
	Preventing worry	‘To avoid creating concern, carrier screening tests should only be offered to risk populations'	2	3.2
Not offering carrier screening at all (*n*=5; 3,4%)				

a Total number of reasons might be higher than *n*=78 and *n*=62; respondents were allowed to give more than one answer.
