# Supplementary material for: Do people from the Jewish community prefer ancestry-based or pan-ethnic expanded carrier screening?
Source: Eur J Hum Genet. 2015 May 13;24(2):171–7. doi: 10.1038/ejhg.2015.97 (PMC4717216; doi:10.1038/ejhg.2015.97)
Supplement: Supplementary Information [file ejhg201597x1.pdf]

Questionnaire

Your opinion about genetic testing with a carrier test for the Jewish Community

VU Medical Center and Academic Medical Center Amsterdam are considering offering a carrier test for relatively common severe genetic diseases that occur in the Ashkenazi Jewish population.

Aim of carrier testing
In the Netherlands, although most children are born healthy, there is a small chance of a child being born with a serious disease. A carrier test can identify, preferably before a woman becomes pregnant, whether there is an increased risk of having a child with a genetic disease. This is determined by testing the potential parents.

Why this questionnaire?
Carrier tests for relatively common severe genetic diseases occurring in the Jewish population are already offered abroad. By means of this questionnaire, VUMC and AMC would like to find out if such tests also need to be offered in the Netherlands, and if so, how.

In this questionnaire we ask about *your experience* with carrier tests and *your opinion* about a carrier test which is specifically targeted on the Jewish community.

Completing the questionnaire will take approximately 15 minutes. The results will be **processed anonymously** into a research report.

If you have any questions about the questionnaire please contact:
Kim Holtkamp, MSc: 020-4449854 or k.holtkamp@vumc.nl

Thank you for your cooperation!

© 2014, Kim Holtkamp, VU University Medical Center, Amsterdam

ALL RIGHTS RESERVED. No part of this questionnaire may be reproduced or transmitted in any form or by any means, electronically or mechanically, including photocopy, recording or any other information storage or retrieval system, without permission in writing from the author.

Clarification for filling out the questionnaire

- Tick the box that most closely resembles **your opinion**.
- Do not think too long. The first reaction is often the best.
- Tick the box that you think reflects your opinion best (unless stated otherwise). For example:

  X Correct

  ☐ Incorrect

- Some questions have the answer option ‘other, please specify…..’. You can fill in your answer if it is not given in the printed answers.
- Filling out the questionnaire will take approximately 10-15 minutes.

If you have any remarks about the questionnaire, please let us know by writing your comments on the last page of this questionnaire.

Background information

What is a carrier?

Everyone is ‘carrier’ of one or more genetic diseases. Since these genetic diseases often do not occur in the family, people are not aware that they are carriers of this disease. When someone is a carrier of a certain disease, he or she does not experience symptoms and will never have the disease. However, when both parents are carriers of the *same* genetic disease, the couple has an increased risk of 1 in 4 (25%) of having an affected child, in every pregnancy.

What is a carrier test?

A carrier test can, preferably before the woman becomes pregnant, identify whether there is an increased risk of having a child with a genetic disease. Parents who are both carriers of the same disease have other reproductive options, besides accepting the risk of a sick child, for instance: examining during the pregnancy if the unborn child is affected. Sometimes it is possible to become pregnant through in vitro fertilization (IVF), during this procedure a healthy embryo is selected and placed in the womb (embryo selection). It is also possible to decide not to have any (more) children.

How common is it that both parents are carriers?

In the Netherlands, certain rare genetic diseases occur in different groups more often than in the general population. This applies to, for example, hereditary anaemia (sickle cell disease) in people with African, Antillean, Surinamese, and/or Mediterranean background, and cystic fibrosis in people of European descent.

What does this mean for the Jewish community?

Within the of origin (Ashkenazi) Jewish community several genetic diseases are more common than in the general population, for example **Tay-Sachs and Canavan disease**. These are metabolic diseases that can cause the deaths of children at a (very) young age.

In the questionnaire, we sometimes refer to ‘severe genetic diseases’. By this, we mean diseases such as Tay-Sachs disease and Canavan disease.

More information about the genetic diseases can be found at: www.erfelijkheid.nl

A. Genetic testing with a carrier test

A carrier test (genetic testing) can identify whether there is an increased risk of having a child with a severe genetic disease. Carrier tests are available for diseases (for instance Tay-Sachs disease or Canavan disease), that are relatively common in the Jewish community.

	Question	Answer
**1.**	Had you ever heard of a carrier test for diseases relatively common in the Jewish community, **before** you received this questionnaire?	☐ No,<br>☐ Yes, via<br>*(multiple answers possible)*<br>☐ Family members<br>☐ Friends/acquaintances<br>☐ General practitioner/family physician<br>☐ Midwife<br>☐ Rabbi<br>☐ Television/radio<br>☐ Newspaper<br>☐ Internet<br>☐ I cannot remember<br>☐ Other, please specify ……………….....................
**2.**	Have you had a carrier test for a disease relatively common in the Jewish community? *(e.g. Tay-Sachs or Canavan)*	☐ No *(Go to question 4))*<br>☐ Yes, in:<br>☐ The Netherlands<br>☐ Belgium<br>☐ United Kingdom<br>☐ United States of America<br>☐ Israel<br>☐ Other, please specify…..………………………….....
**3.**	If you **have** been tested, how did you have the test done?	☐ In a hospital (Outpatient clinic for genetic testing)<br>☐ Via Dor Yeshorim (Anonymous screening programme)<br>☐ Via (secondary) school<br>☐ Requested via the internet<br>☐ I cannot remember how<br>☐ I have not been tested<br>☐ Other, please specify.………………………………...

**4.**	Do you know someone or have you known someone with a severe **genetic** disease?	☐ No<br>☐ Yes, please specify the disease……………………………in<br>☐ family member<br>☐ Friend/acquaintance<br>☐ Other, please specify… ………………………...

B. Your opinion about carrier tests in the Jewish community

Please indicate to what extent you agree with the following statements. There are no right or wrong answers, **we are interested in your opinion.**

		Strongly disagree	Disagree	Undecided	Agree	Strongly agree
**1.**	Offering a carrier test **avoids much suffering.**	☐	☐	☐	☐	☐
**2.**	A carrier test gives couples **more certainty** about their risk of having an affected child.	☐	☐	☐	☐	☐
**3.**	Offering a carrier test **leads to anxiety** in the Jewish community.	☐	☐	☐	☐	☐
**4.**	Offering a carrier test can cause people to **feel forced** to get tested.	☐	☐	☐	☐	☐
**5.**	Carrier testing will lead to carriers feeling **left out** of the Jewish community.	☐	☐	☐	☐	☐
**6.**	Offering a carrier test specifically aimed at the Jewish community leads to **discrimination** of Jews.	☐	☐	☐	☐	☐
**7.**	The results of a carrier test can **help couples in making decisions** about having children.	☐	☐	☐	☐	☐
**8.**	The results of a carrier test can help in **choosing a partner**.	☐	☐	☐	☐	☐

**9.**	When both parents are carriers, **examination of the foetus during the pregnancy** is acceptable.	☐	☐	☐	☐	☐
**10.**	When both parents are carriers, conceiving via **in vitro fertilization (IVF) and embryo selection** is acceptable.	☐	☐	☐	☐	☐
**11.**	When both parents are carriers, **termination of a pregnancy when the child is affected**, is acceptable.	☐	☐	☐	☐	☐
**12.**	Every Jewish couple who wants to have children should have the **option** to have a carrier test.	☐	☐	☐	☐	☐
**13.**	Every Jewish couple who wants to have children **must** have a carrier test.	☐	☐	☐	☐	☐
**14.**	Health care professionals are allowed **to insist** couples who want to have children to have a carrier test.	☐	☐	☐	☐	☐

For the following questions different words with their opposite meaning are listed. Tick the box that most closely resembles your opinion. You can choose the middle box if your opinion is neutral.

Example: 'When choosing a holiday destination, the weather at the destination is …'
Suppose, you find the weather at the destination a little bit important when choosing a holiday destination, than you fill in the question as followed.

**Unimportant**	☐	☐	☐	**X**	☐	**Important**

15. "Offering carrier tests specifically aimed at severe genetic diseases that are more common in the Jewish Community is …"

Bad	☐	☐	☐	☐	☐	Good
Not alarming	☐	☐	☐	☐	☐	Alarming

Desirable	☐	☐	☐	☐	☐	Not desirable
Not self-evident	☐	☐	☐	☐	☐	Self-evident

C. Your opinion about a carrier test, if you had the choice yourself

A carrier test (genetic testing) can identify whether there is an increased risk of having a child with a severe genetic disease. Carrier tests are available for diseases (for instance Tay-Sachs disease or Canavan disease), that are relatively common in the Jewish community.

**1.**	Would you have a carrier test yourself?	☐ Certainly<br>☐ Probably<br>☐ Maybe/maybe not<br>☐ Probably not<br>☐ Certainly not
**2.**	Why would you (not) have a carrier test?	………………………………………………………………………
**3.**	I am worried about my own risk of being a carrier of a severe genetic disease.	☐ Strongly disagree<br>☐ Disagree<br>☐ Undecided<br>☐ Agree<br>☐ Strongly agree
**4.**	Do you have a partner?	☐ Yes<br>☐ No *(Go to question 7)*
**5.**	Do you and your partner want to have children?	☐ Yes<br>☐ Maybe<br>☐ No *(Go to question 7)*<br>☐ Inapplicable

Suppose you and your partner are both carrier of the same severe genetic disease, and you therefore have an increased risk (25%) of having a child with a severe genetic disease.

6. To what extent do you agree with the following statements?

		Strongly disagree	Disagree	Undecided	Agree	Strongly disagree
**a.**	We would take the risk and not take any action (the child is born as he or she is).	☐	☐	☐	☐	☐
**b.**	We would decide not to have (more) children.	☐	☐	☐	☐	☐
**c.**	We would consider examination of the foetus during the pregnancy (prenatal testing by chorionic villus sampling).	☐	☐	☐	☐	☐
**d.**	We would consider a test tube fertilization (IVF) with embryo selection.	☐	☐	☐	☐	☐
**e.**	We would consider termination of the pregnancy if the child has been affected.	☐	☐	☐	☐	☐

Suppose you have the option to have a carrier test.

7. To what extent is the opinion of others important in making this decision?

	Unimportant	Somewhat unimportant	Undecided	Somewhat important	Important	Inapplicable
a) My general practitioner/family physician	☐	☐	☐	☐	☐	☐
b) My midwife	☐	☐	☐	☐	☐	☐
c) My partner	☐	☐	☐	☐	☐	☐
d) My family	☐	☐	☐	☐	☐	☐
e) My rabbi	☐	☐	☐	☐	☐	☐
e) Other, please specify	....					

8. Which characteristics of a carrier test are important for your decision to have or not have a carrier test?

Rank the following characteristics in order of importance from 1 to 5. **Give a 1 to the most important characteristic, and a 5 to the least important characteristic.** Rank the remaining characteristics as a 2,3 or 4 in order of importance. Please use each number only once.

		Importance
**a.**	Time of results of the test (how long it takes before the results are available).	☐
**b.**	Reliability of the test (how certain it is that the outcome is correct).	☐
**c.**	Costs (of the test).	☐
**d.**	Travel distance (to the location of testing).	☐
**e.**	Waiting time (how soon can I get tested).	☐

D. Which diseases should be tested?

Within the Jewish community there are several diseases which are relatively more common than in the general population. These diseases differ in severity and the frequency with which they occur. The following questions are designed to establish what kind of diseases you think should be included in the carrier test.

For what kind of diseases should a carrier test be offered?

		Strongly disagree	Disagree	Undecided	Agree	Strongly disagree
**a.**	Serious, life-threatening diseases for which no treatment is available.	☐	☐	☐	☐	☐
**b.**	Diseases involving severe mental disability.	☐	☐	☐	☐	☐
**c.**	Diseases involving a severe physical disability (such as wheelchair-dependence).	☐	☐	☐	☐	☐

d.	Severe diseases that occur later in life (such as breast cancer)	☐	☐	☐	☐	☐
e.	All diseases that a couple wants to be tested on.	☐	☐	☐	☐	☐
f.	Other, please specify	…………………………………………………………………………....				

E. Offering of a carrier test for the Jewish community

We would like to hear your opinion on how a carrier test should best be offered.

**1.**	To whom should a carrier test best be offered?<br>*(Multiple answers possible)*	☐ To school pupils at (Jewish) secondary school<br>☐ To students during university/college<br>☐ To couples before engagement/marriage<br>☐ To couples before pregnancy<br>☐ To the expectant parent(s) during pregnancy<br>☐ Newborns<br>☐ I think that a carrier test should not be offered<br>☐ Other, please specify………………………………….
**2.**	How should a carrier test best be offered?<br>*(Multiple answers possible))*	☐ Via the hospital (Outpatient clinic for genetic testing)<br>☐ Via the general practitioner/family physician<br>☐ Via the midwife<br>☐ Via Dor Yeshorim (Anonymous screening programme)<br>☐ Via (Jewish) secondary school<br>☐ Via the rabbi<br>☐ Via the internet<br>☐ I think that a carrier test should not be offered.<br>☐ Other, please specify……….…………………….
**3.**	*Suppose a couple that wants to have children has had a carrier test.*<br>How should the results of the carrier test be given?	☐ **Individually:** both partners get their own results and receive information about which diseases they are a carrier of.<br>☐ **As a couple**: the couple receives information about whether they are both carriers of the same disease or not (the couple does not receive separate results per person)<br>☐ I have no preference<br>☐ Other, please specify….…………………………..

**4.**	If a carrier test for **various diseases** is being offered, how should it be offered?	☐ **All or nothing**: the list of diseases that can be tested on is closed. The decision to have a carrier test means that the person will be tested on all those diseases.<br>☐ **Bundle**: The list of diseases is divided into groups. A group consists of diseases that are similar in type and severity. The person can choose which groups of diseases he/she wants to be tested on.<br>☐ **Free choice:** the person that wants to be tested can chose from a list of diseases on which ones he/she wants to be tested on.<br>☐ Other, please specify…..…………………………..
**5.**	If a carrier test is offered, who should pay for it?	☐ The health insurance<br>☐ Partly the individual/ partly the health insurance<br>☐ The individual him/herself
**6.**	How much would you be willing to pay for a carrier test?	☐ Less than €50<br>☐ €50- €100<br>☐ €100- €200<br>☐ €200 - €500<br>☐ €500 - €1000<br>☐ €1000 - €1500<br>☐ €1500 - €2000<br>☐ More than €2000

F. Offering a carrier test to <u>everyone</u>

The aforementioned genetic diseases such as Tay-Sachs and Canavan disease occur particularly in the Jewish community. However, there are more genetic diseases than just these two examples. Moreover, non-Jewish people may also give birth to a baby with a severe genetic disease.

Which do you prefer:

- ☐ **Each subpopulation receives a different carrier test** which only tests for the common diseases in this group.
- ☐ Regardless of their origin, **everyone in the Netherlands** is offered the same carrier test which tests for all possible genetic diseases.

Please explain your answer:

………

………

………

G. General information

Finally, we would like some general information about you.

**1.**	On what date did you filled out this questionnaire?	|____|____| day |____|____| month |_2_|_0_|___|____| year
**2.**	How old are you?	………… years old
**3.**	What is your gender?	☐ Male<br>☐ Female
**4.**	What are the first two digits of your postcode (in the Netherlands)?	☐☐XX
**5.**	What is your highest level of completed education?	☐ Elementary school/ none<br>☐ Lower level of secondary school<br>☐ Lower vocational training<br>☐ Intermediate vocational training<br>☐ Higher level of secondary school<br>☐ Higher vocational training/university<br>☐ Other, please specify………………………………..……………
**6.**	What is the country of birth of:	Yourself..………………………………………………………............<br>Your mother ….……………………………….………………………..<br>Your father…….………………………………………………………..
**7.**	Are you of:	☐ Ashkenazi origin<br>☐ Sephardic origin<br>☐ Partly Ashkenazi/Partly Sephardic origin<br>☐ I do not know<br>☐ I am not Jewish<br>☐ Other, please specify…………………………………………..
**8.**	What is your religious affiliation	☐ Ultra-Orthodox Judaism<br>☐ (Modern) Orthodox Judaism<br>☐ Liberal Judaism<br>☐ Other, please specify………………………………………….<br>☐ None
**9.**	How active are you in your religion?	☐ Very active<br>☐ Somewhat active<br>☐ Not active<br>☐ Inapplicable

**10.**	What is your living situation?	☐ Married/Living together with a Jewish partner<br>☐ Married/ Living together with a non-Jewish partner<br>☐ Single<br>☐ Other, please specify……………………………………….
**11.**	Do you have children (of your own)?	☐ Yes: ……… (number)<br>☐ No

For comments/suggestions, please use the space below:

…………………………………………………………………………...………………………………

…………………………………………………..…………………………………………………………

Thank you for completing the questionnaire
